# A Point‐of‐Care Model for Hepatitis C Elimination in Remote Islands of Taiwan

**DOI:** 10.1002/kjm2.70060

**Published:** 2025-06-06

**Authors:** Tzu‐Chun Lin, Pei‐Chien Tsai, Chung‐Feng Huang, Ming‐Lun Yeh, Yu‐Ju Wei, Ming‐Yen Hsieh, Ming‐Jong Bair, Chia‐Yen Dai, Jee‐Fu Huang, Ming‐Lung Yu, Wan‐Long Chuang

**Affiliations:** ^1^ Hepatobiliary Division, Department of Internal Medicine Kaohsiung Medical University Hospital, Kaohsiung Medical University Kaohsiung Taiwan; ^2^ Center of Excellence for Metabolic Associated Fatty Liver Disease National Sun Yat‐sen University Kaohsiung Taiwan; ^3^ Hepatitis Center Kaohsiung Medical University Hospital, Kaohsiung Medical University Kaohsiung Taiwan; ^4^ Hepatitis Research Center Kaohsiung Medical University Kaohsiung Taiwan; ^5^ Center for Metabolic Disorders and Obesity Kaohsiung Medical University Kaohsiung Taiwan; ^6^ Division of Gastroenterology, Department of Internal Medicine Taitung Mackay Memorial Hospital Taitung Taiwan; ^7^ Mackay Medical College New Taipei City Taiwan

**Keywords:** elimination, hepatitis C virus, point‐of‐care, under‐resourced islands

## Abstract

Timely and efficient diagnosis of hepatitis C virus (HCV) infection remains the effective approach for the subsequent care cascade of HCV treatment. It is of importance in under‐resourced areas. The study aimed to assess the feasibility of a point‐of‐care (POC) model by a rapid diagnostic test and subsequent confirmational HCV RNA test in remote islands where traffic is an additional hurdle for health care. We conducted a mass POC screening program in 3 outlying islands, including Liuqiu (6.8 km^2^, 12,000 residents), Green (15.1 km^2^, 4280 residents), and Orchid (48.4 km^2^, 5230 residents) islands. We used immunochromatography‐based finger‐tip assays for HCV antibody detection. Serum HCV RNA was measured among patients seropositive for the rapid anti‐HCV test. There were 1055, 268, and 276 adult residents receiving rapid tests in Liuqiu, Green, and Orchid, respectively, yielding response rates of 47.0%, 41.1%, and 24.4%, respectively. The prevalence of anti‐HCV‐positive were 1.3% (*n* = 14), 1.1% (*n* = 3), and 0, respectively. Nine (52.9%) of the 17 anti‐HCV‐positive patients were HCV RNA‐negative. The HCV RNA‐positive patients then received anti‐viral treatment. The average turnaround time for overall POC HCV test results was 11.8 ± 3.2 min, and the sampling time was completed within 10–30 s. Meanwhile, the labor cost of HCV RNA screening was 156.3 USD, which was a 28.6% decrease compared to traditional methods. The study demonstrated the feasibility and effectiveness of the POC model for HCV elimination in remote islands with limited resources.

**Abbreviations**
CHC: chronic hepatitis C
DAAs: direct antivirals
HBV: hepatitis B virus
HCC: hepatocellular carcinoma
HCV: hepatitis C virus
POC: point‐of‐care
RDT: rapid diagnostic test
SVR: sustained virological response
WHO: World Health Organization

## 1 Introduction

Hepatitis C virus (HCV) infection is a major issue of global public health. It is estimated that approximately 71 million people worldwide suffer from chronic hepatitis C (CHC). CHC leads to liver complications such as cirrhosis, hepatocellular carcinoma, and end‐stage liver disease [1]. In addition, 70%–75% of patients may develop extrahepatic manifestations, contributing to increased mortality. All of which impose tremendous health and economic burdens globally [2, 3, 4]. The public health issue is underestimated mainly because of low disease awareness and the lack of effective preventive measures [5]. With the development of direct‐acting antivirals (DAAs), the cure rate for HCV has reached 97%, providing long‐term prevention of chronic HCV infection outcomes [6]. This has significantly impacted the reduction of new infections by 90% and HCV‐related deaths by 65%. This changing landscape of treatment contributes substantially towards achieving the World Health Organization goal of eliminating HCV by 2030 [7]. However, the current directions for HCV elimination in large part depend on a case‐finding approach and micro‐elimination of special populations [8]. The efforts might be much affected in the under‐resourced areas where the shortage of medical staff and delivery of healthcare resources were the major hurdles. Without efforts to improve strategy for both screening and linkage‐to‐care aspects, treatment alone may not be sufficient to achieve the goal of HCV elimination [9].

Taiwan has a high prevalence of CHC. The estimated nationwide prevalence is 4%–9%, and the prevalence exceeds 20% in some hyperendemic areas [10]. Several measurements have been vigorously implemented over the past decade to achieve the ultimate goal of eliminating HCV. These efforts include amelioration of the epidemic in hyperendemic areas and treatment programs for high‐risk groups such as CHC patients undergoing dialysis, people who inject drugs, patients with psychiatric disorders, and prisoners [11, 12, 13, 14]. The implementation of HCV reflex testing strategies in hospitals and in‐hospital micro‐elimination projects also helped to mitigate the epidemic of HCV infection [4, 15, 16].

Besides increasing disease awareness, educational and screening programs are key determinants for overcoming the hurdles of the elimination of HCV [4, 16, 17, 18]. The limited medical resources in countryside areas, especially in remote islands, deserve more attention in public health. Recently, the use of rapid HCV antibody tests, a simplified version of laboratory testing, possesses the potential to overcome major barriers to testing in various settings. It provides results immediately or within a short period. Using fingerstick blood samples for point‐of‐care (POC) HCV testing eliminates the need for laborious venipuncture by increasing accessibility and saving time and cost [19, 20]. POC HCV screening played an important role in specific populations, including people who use drugs, incarcerated individuals, people living with HIV, residents of halfway houses, as well as in emergency departments and community pharmacies. These efforts have successfully identified hidden HCV populations and effectively linked them to the HCV care cascade [21, 22]. This approach also effectively reduces the need for blood collection personnel and increases patient willingness to participate in screening. However, the application of the POC measure has rarely been investigated in the under‐resourced areas.

Consequently, this study aimed to evaluate the feasibility of a POC model for HCV screening and linkage‐to‐care in Taiwan's remote islands. The turnaround time and labor costs between POC and traditional methods were also compared.

## 2 Methods

### 2.1 Study Design, Setting, and Subjects

The Institutional Review Board of Kaohsiung Medical University Hospital approved this study before it was performed. The study was performed in accordance with the ethical standards in the Helsinki Declaration of 1975, as revised in 2008. We invited all the adult residents participating in the HCV screening campaign in Liuqiu, Green, and Orchid islands from February to August 2023. All subjects provided written informed consent.

### 2.2 Geographic and Medical Care Characteristics (Figure 1)

**FIGURE 1:**
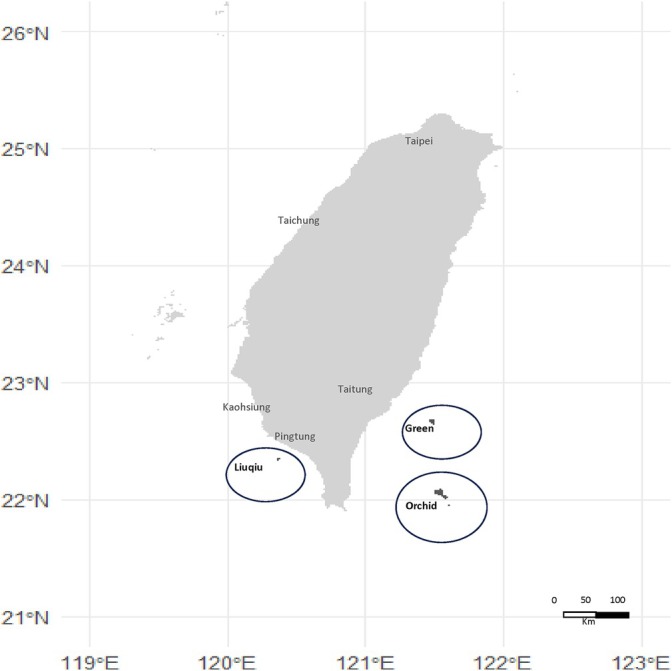
The locations of remote islands in Taiwan.

#### 2.2.1 Liuqiu

Located southwest of Pingtung County, Taiwan, Liuqiu Island is an offshore island with a total area of 6.8 km^2^ and a population of 12,000 residents. The health center is the only equipped medical facility with two medical doctors on the island, serving dual roles in public health and medical care.

#### 2.2.2 Green Island

Situated in the Pacific Ocean southeast of Taitung County, Taiwan, Green Island has a total area of 15.1 km^2^ and a population of 4280 residents. The health center is the only medical institution with two medical doctors providing 24‐h healthcare services.

#### 2.2.3 Orchid Island

Located south of Green Island, Orchid covers an area of 48.4 km^2^ with a population of approximately 5230 people, most of whom belong to the Indigenous Tao tribe. With only one medical doctor, the health center is the sole provider of medical, emergency, and public health services on the island, safeguarding the health and lives of Orchid's residents around the clock.

### 2.3 Demographic and Follow‐Up Data

All participants received hepatitis education, completed a health questionnaire assessment, and underwent POC HCV screening. The health questionnaire collected data on age, gender, ethnicity, education, employment status, and risk factors for HCV infection. The educational sessions included information on the risk factors for HCV transmission, the progression of liver disease, complications of HCV infection, and available treatments. On‐site evaluations were conducted by a team comprising two specialists, several nurses, and researchers who facilitated the health questionnaire assessment and POC HCV screening. All participants testing positive for HCV underwent additional testing for HCV viral load and abdominal ultrasound examinations.

### 2.4 Laboratory Examination

The POC HCV screening used the Toyo Anti‐HCV Rapid Antibody Test, a rapid chromatographic immunoassay (Türklab Medical Devices, Izmir, Turkey) for the qualitative detection of antibodies produced against proteins encoded by conserved sequences of the HCV genome in human serum, plasma, and whole blood samples. The test demonstrated a sensitivity of 98.0% and a specificity of 99.0% [23]. All blood collection procedures were performed by professionally trained nurses. Participants' fingertips were cleaned and pricked with a sterile lancet to collect 30 μL of blood, which was applied to the test cassette for POC HCV screening. Results indicating the presence of HCV antibodies were available within 15 min. POC HCV screening seropositive samples were subsequently collected for antibodies to anti‐HCV and HCV RNA testing via blood testing. Anti‐HCV antibodies were detected and rechecked using a commercially available chemiluminescent microparticle immunoassay (CMIA; Abbott Architect i‐1000 system, Abbott Diagnostics, Lake Forest, Illinois, USA). HCV virology blood samples were subsequently collected for viral load testing. HCV serology was assessed using a standardized automated qualitative reverse transcription polymerase chain reaction (RT‐PCR) assay to measure quantitative serum HCV RNA levels, with a detection limit of 30 IU/mL [24]. Finally, experienced hepatology specialists conducted abdominal ultrasound examinations for disease severity and HCC surveillance.

### 2.5 Statistical Analysis

The characteristics of study participants were presented as numbers (proportions) for categorical data and as mean ± standard deviation for continuous data. Pearson's chi‐square test and Fisher's exact test were used to appropriately test the association between categorical variables, and an independent sample t‐test was used to examine continuous variables. All statistics used a two‐tailed significance test of *p* < 0.05. A two‐tailed significance level of *p* < 0.05 was considered statistically significant for all analyses. Statistical analyses were conducted using SPSS version 20.0 (IBM Corp., Armonk, NY, USA), and GraphPad Prism version 9.0 was used for plotting.

## 3 Results

**FIGURE 2:**
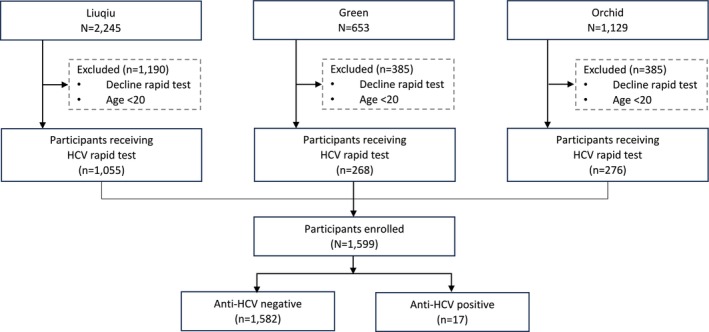
Study flowchart.

A total of 11 screening campaigns were conducted during the study period, inviting 2245 adult residents of Liuqiu, 653 of Green, and 1129 of Orchid, respectively. Among these, 2425 subjects (59.8%) were excluded due to refusal to participate, incomplete examinations, or not meeting the age criteria. Totally, 1055 of Liuqiu, 268 of Green, and 276 of Orchid adult residents received rapid testing, yielding response rates of 47.0%, 41.1%, and 24.4%, respectively (Figure 2).

Table 1 shows the demographic characteristics of the subjects. The average age of participants in the rapid HCV test was 60.3 ± 11.6 years old, and 51.9% were females. A total of 16.8% (*n* = 268) subjects were illiterate. The prevalence of hypertension, diabetes, and chronic kidney disease was 38.5%, 20.1%, and 1.5%, respectively. HCV risk factors included an HCV infection of a family member (0.8%), surgery (61.2%), blood transfusions (7.2%), acupuncture (4.3%), ear piercing or tattooing (13.6%), and dental treatment by unlicensed practitioners. The prevalence of anti‐HCV positivity by POC testing was 1.1% (*n* = 17), including 1.3% (*n* = 14) in Liuqiu, 1.1% (*n* = 3) in Green, and 0% in Orchid, respectively. The average turnaround time for POC HCV test results was 11.8 ± 3.2 min: 12.7 ± 2.7 min in Liuqiu, 12.0 ± 3.4 min in Green, and 10.3 ± 3.0 min in Orchid. Univariate analysis revealed that the significant risk factor for anti‐HCV+ was a history of blood transfusion (Table 2).

**TABLE 1:** Demographic characteristics of screened participants.

Characteristics	All	Liuqiu	Green	Orchid
N	1599	1055	268	276
Age, years	60.3 ± 11.6	61.9 ± 10.6	55.6 ± 13.1	58.7 ± 11.9
≥ 60	917 (57.3)	673 (63.8)	107 (39.9)	137 (49.6)
Sex				
Females	830 (51.9)	576 (54.6)	129 (48.1)	125 (45.3)
Education				
Illiteracy	268 (16.8)	237 (22.5)	21 (7.8)	10 (3.6)
Elementary school	491 (30.7)	360 (34.1)	57 (21.3)	74 (26.8)
High school	705 (44.1)	392 (37.1)	151 (56.3)	162 (58.7)
College or above	135 (8.4)	66 (6.3)	39 (14.6)	30 (10.9)
Smoking	291 (18.2)	146 (13.8)	64 (23.9)	81 (29.4)
Alcohol	286 (17.9)	132 (12.5)	51 (19.0)	103 (37.3)
Hypertension	616 (38.5)	429 (40.7)	109 (59.3)	78 (28.3)
Diabetes	322 (20.1)	252 (23.9)	39 (14.6)	31 (11.2)
Chronic kidney disease	24 (1.5)	21 (2.0)	1 (0.4)	2 (0.7)
Risk factors of HCV				
HCV‐infected family members	12 (0.8)	10 (1.0)	2 (0.8)	0 (0.0)
Surgery	978 (61.2)	674 (63.9)	155 (57.8)	149 (54.0)
Transfusion	115 (7.2)	71 (6.7)	17 (6.3)	27 (9.8)
Tattoos/piercings/acupuncture	686 (4.3)	416 (39.4)	127 (47.4)	143 (51.8)
Unlicensed dental treatment	218 (13.6)	174 (16.5)	27 (10.1)	17 (6.2)
POC Test				
Anti‐HCV+	17 (1.1)	14 (1.3)	3 (1.1)	0 (0.0)
HCV RNA+	8 (47.1)	6 (42.9)	2 (66.7)	0 (0.0)
Turnaround time	11.8 ± 3.2	12.7 ± 2.7	12.0 ± 3.4	10.3 ± 3.0

*Note*: Values expressed as mean ± standard deviation or sample size and proportion (%).

Abbreviations: HCV, hepatitis C virus; POC, point‐of‐care.

**TABLE 2:** Characteristics of the participants with anti‐HCV tests.

Characteristics	HCV negative (*n* = 1582, 98.9%)	HCV positive (*n* = 17, 1.1%)	*p* value
Age, years	60.3 ± 11.6	57.2 ± 11.5	0.274
≥ 60	910 (57.5)	7 (41.2)	0.175
Sex			
Females	822 (52.0)	8 (47.1)	0.688
Education			
Illiteracy	265 (16.8)	3 (17.7)	0.063
Elementary school	490 (31.0)	1 (5.9)	
High school	693 (43.8)	12 (70.6)	
College or above	134 (8.4)	1 (5.6)	
Smoking	286 (18.1)	5 (29.4)	0.228
Alcohol	282 (17.8)	4 (23.5)	0.525
Hypertension	607 (38.4)	9 (52.9)	0.219
Diabetes	318 (20.1)	4 (23.5)	0.726
Chronic kidney disease	24 (1.5)	0 (0.0)	1.000
Risk factors of HCV			
HCV‐infected family members	12 (0.8)	0 (0.0)	1.000
Surgery	970 (61.3)	8 (47.1)	0.230
Transfusion	111 (7.0)	4 (23.5)	0.029
Tattoos/Piercings/Acupuncture	678 (42.9)	8 (47.1)	0.728
Unlicensed dental treatment	216 (13.7)	2 (11.8)	1.000

*Note*: Values expressed as mean ± standard deviation or sample size and proportion (%).

Abbreviation: HCV, hepatitis C virus.

**FIGURE 3:**
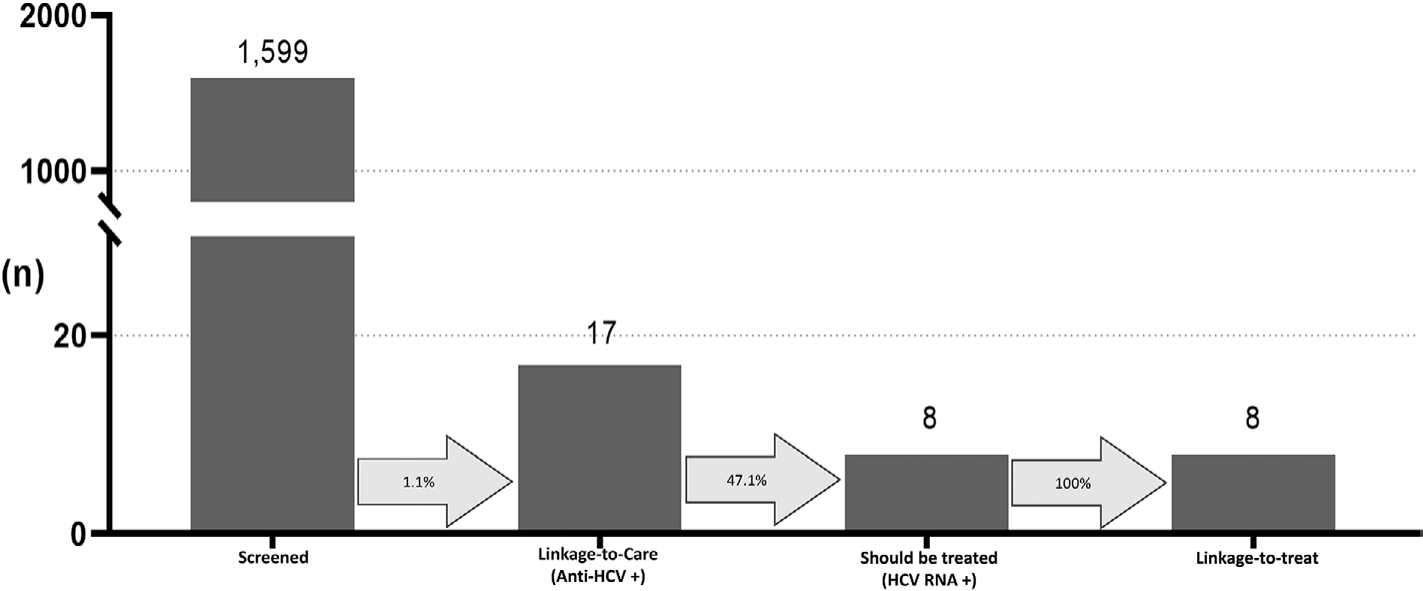
HCV care cascades of the study.

Seventeen anti‐HCV+ patients received further HCV RNA testing and the results were shown within 2 days (Table 3). Among them, eight patients were HCV RNA+ and subsequently received DAA treatment (Figure 3).

**TABLE 3:** Details of POC HCV tests with positive results.

No.	POC HCV test results (minute)	Anti‐HCV	HCV‐RNA test results (day)	HCV‐RNA
01	11.7	Positive	2	Positive
02	15.2	Positive	2	Negative
03	10.1	Positive	2	Negative
04	12.3	Positive	2	Negative
05	10.4	Positive	2	Positive
06	11.1	Positive	2	Negative
07	14.7	Positive	2	Negative
08	11.8	Positive	2	Negative
09	10.4	Positive	2	Negative
10	9.5	Positive	2	Positive
11	9.8	Positive	2	Negative
12	9.0	Positive	2	Positive
13	9.8	Positive	2	Positive
14	10.2	Positive	2	Positive
15	9.2	Positive	2	Negative
16	10.0	Positive	2	Positive
17	11.5	Positive	2	Positive

Abbreviations: HCV, hepatitis C virus; POC, point‐of‐care.

**FIGURE 4:**
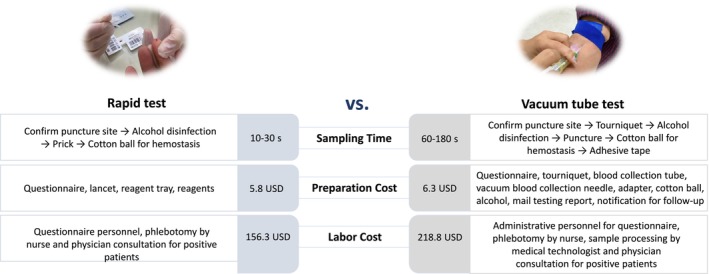
Comparison of sampling times and costs of the rapid test and the vacuum tube test.

The time and cost of using rapid tests and vacuum tube tests at screening sites are shown in Figure 4. The rapid test takes approximately 10–30 s to perform, while the vacuum tube spot check takes approximately 60–180 s. The rapid test has less cost spent on medical material preparation. Similarly, the labor cost of each POC anti‐HCV screening test was USD 156.3, which was less than the USD 218.8 of the traditional method (a decrease of 28.6%). In addition, compared to the conventional vacuum tube blood collection method, rapid testing only needed about two drops of fingertip blood, with a shorter collection time. Results were available within 15 min, and the smaller collection wound was less invasive. Rapid testing also had fewer location constraints, making it more adaptable. Since the procedure caused minimal pain from a single finger prick, participants were more willing to undergo testing and it was relatively easier for medical staff to collect the test (Table S1).

## 4 Discussion

Despite tremendous achievements developed in HCV diagnosis and treatment during the past decade, the undiagnosed patients remain a significant challenge in the path of HCV elimination [25]. The study demonstrated the feasibility of a POC model for HCV screening in the remote islands. The model provided moderately high screening rates in the remote islands, where the shortage of medical staff and facilities is common. We demonstrated that the POC model provided an efficient and easy‐to‐access approach for the HCV patient care cascade in the under‐resourced areas. The POC rapid test saved a more significant turnaround time and labor cost than traditional methods, which was an excellent model for HCV care [7].

Rapid HCV antibody test demonstrated excellent diagnostic performance across diverse settings and populations [24]. Rapid HCV antibody test not only provided results within a short time frame, but also minimized the need for healthcare workers to perform fingerstick procedures. It therefore enhanced patient's willingness to undergo screening and reduced occupational hazards [26]. The study also tackled health inequities caused by geographical barriers, and significantly improved healthcare accessibility for residents of remote islands. All of which may increase screening coverage rates and improve the efficiency for patient care cascades.

The complexity of past HCV screening and diagnostic processes could be a main hurdle for the HCV care cascade. It includes the need to confirm exposure with HCV antibody testing, confirm active infection with HCV RNA testing, and conduct one or more assessments before initiating treatment [27]. Therefore, an easy‐to‐access model will much lower the ban for initial screening and diagnosis gates. Hsiang et al. in a halfway house demonstrated that direct access to POC HCV screening improved linkage to care and simplified the HCV care cascade, resulting in increased treatment uptake. A study by Howell et al. showed that a simplified, nurse‐led model of care based on POC HCV testing, delivered through a needle and syringe exchange program, successfully engaged participants in HCV testing, with high rates of subsequent care engagement and treatment uptake [21]. Our findings demonstrated moderately high screening rates (24.4%–47.0%) in the remote islands, where the medical staff and facilities are limited. Moreover, compared to traditional methods, the overall screening cost for POC HCV testing was reduced by 62.5 USD.

A key feature of this study is that POC HCV testing was provided and conducted by on‐site healthcare personnel within the community. The use of on‐site testing facilitated the entire screening process carried out at a convenient location in a community setting. Our results echoed previous studies showing that rapid POC HCV testing can be performed with a turnaround time of less than 15 min and reduces costs. Traditional venipuncture services have significant barriers related to venous access, needle phobia, and turnaround time for results. O'Loan et al. demonstrated that the incorporation of HCV POC testing in community and outreach settings is critical to reducing barriers in HCV diagnosis. The rapid turnaround of HCV RNA results ensured faster initiation of treatment and avoided the risk of loss to follow‐up [28]. Koo et al. demonstrated that compared to standard care screening, POC HCV testing resulted in an additional 0.035 QALY per person and reduced costs by 21.15 USD, proving to be both cost‐effective and cost‐saving [25]. Shackman et al. revealed that on‐site rapid HCV testing had an ICER of 18,300 USD/QALY compared to no testing, making it more effective than off‐site HCV testing referrals [29]. Moreover, integrating rapid POC HCV antibody and RNA testing can optimize detection outcomes, connecting more individuals currently infected with HCV to subsequent treatment [30].

A low screening rate is not uncommon in a community‐based setting. It was in a large part contributed to by the low disease awareness in our community, particularly in the under‐resourced areas [31]. It echoed our previous study showing that low disease awareness was the major hurdle for HCV care cascade [32]. Although our study successfully recruited participants for screening activities in collaboration with primary care centers, the proportion of participants who completed rapid fingerstick blood testing remained low (24.4%–47.0%). It suggested that multiple barriers to actual implementation remained. Possible reasons for low screening rates included low disease awareness, insufficient knowledge, stigma and discrimination, and impaired medical access of super‐aged community [33]. Therefore, having tight collaboration with local health centers is quite important to achieve the target goal. In addition, integrating follow‐up tracking and referral resources is essential to improve the effectiveness of screening. The screening rate of the POC HCV test could be significantly increased if it were operated by local doctors or nurses with adequate training and supporting logistics.

This study has certain limitations. First, there is a lack of comparative results from multiple POC HCV tests, which may raise concerns about insufficient information. Secondly, there was a lack of information on risk factors for HCV infection, including history of PWID/HIV/prisoners, and therefore, they could not be included in the analysis. Lastly, the aim of the study was to investigate the performance of an efficient and cost‐effective approach in the under‐resourced areas. Therefore, other etiologies of hepatitis such as hepatitis B infection and HCC surveillance were not included in the methods.

In conclusion, this study demonstrated an effective model connecting screening, diagnosis, and treatment in under‐resourced remote islands. The POC test is a valuable tool, offering high efficiency and cost‐effectiveness for HCV screening. Implementing the POC model for localized testing in resource‐limited remote islands is a feasible approach to improve accessibility to rapid HCV screening.

## Disclosure

Potential Competing Interests: Jee‐Fu Huang: Research Grant from Gilead, Bristol‐Myer‐Squibb. Consultant of Roche, Sysmex, Boehringer Ingelheim, and Aligos. Speaker for Abbvie, Gilead, Merck, Sysmex, and Novo Nordisk. Chia‐Yen Dai: Consultant of Abbvie and Roche. Speaker for Abbvie, Gilead, and Roche. Chung‐Feng Huang: Speaker for Abbvie, BMS, Bayer, Gilead, Merck, and Roche. Ming‐Lung Yu: Research grant from Abbott, BMS, Merck, and Gilead; Consultant of Abbvie, Abbott, Ascletis, BMS, Merck, Gilead, and Roche; Speaker for Abbvie, Abbott, BMS, Merck, Gilead, and IPSEN. Wan‐Long Chuang: Consultant of Gilead, AbbVie, BMS, and PharmaEssentia; Speaker for Gilead, AbbVie, BMS, and PharmaEssentia.

## Ethics Statement

The study was approved by Institutional Review Board of Kaohsiung Medical University Hospital. All subjects provided written informed consent.

## Conflicts of Interest

The authors declare no conflicts of interest.

## Supporting information


**Table S1.** Comparison of hepatitis C test methods.

## Data Availability

The data and materials are available in Kaohsiung Medical University Hospital and could be obtained only with the approval of the corresponding author.
