# Supplementary material for: A Point‐of‐Care Model for Hepatitis C Elimination in Remote Islands of Taiwan
Source: Kaohsiung J Med Sci. 2025 Jun 6;41(10):e70060. doi: 10.1002/kjm2.70060 (PMC12520535; doi:10.1002/kjm2.70060)
Supplement: Supplementary file 1 — Table S1. Comparison of hepatitis C test methods. [file KJM2-41-e70060-s001.docx]

**Supplementary Table 1**. Comparison of hepatitis C test methods

| **Rapid test** | **Evaluation** | **Item** | **Evaluation** | **Vacuum tube test** |
| --- | --- | --- | --- | --- |
| This is a single test item. If Hepatitis C antibodies are positive, referral to a medical institution for viral load testing is required. | Few | **Testing Items** | Many | Multiple test items; if Hepatitis C antibodies are positive, viral load testing can be performed directly. |
| No sample submission is required, reducing backend manpower, blood testing, and material costs. | Low | **Manpower** | High | Sample submission is required; it involves laboratory testing and inspection report operations manpower. |
| Approximately 2 drops of blood were collected (using a blood glucose lancet). | Short | **Sampling Time** | Long | 5-7ml of venous blood collected (using standard blood collection tools). |
| Results are available in 15 minutes. | Short | **Testing Time** | Long | Approximately one month. |
| Non-safety lancets, prone to accidental needle pricks. | Low | **Safety** | High | Safety needles were used. |
| Rapid collection with a small wound. | Minimal | **Invasiveness** | High | The needle stays inserted for a longer time, resulting in a larger wound. |
| It can be performed anywhere. | Low | **Location Restrictions** | High | Requires a flat surface and table or chair setup. |
| Tools required: blood glucose lancet, testing tray, reagent, alcohol swab, needle disposal container, garbage bag. | Few | **Required Tools** | Many | Tools required: blood draw pillow, needles, collection tubes, tourniquet, alcohol swabs, adhesive tape, needle disposal container, garbage bag. |
| Single needle prick. | Short | **Pain Duration** | Long | Needle insertion and withdrawal occur twice; potential foreign body sensation during collection (multiple pricks may be needed in difficult cases). |
| Quick process, fast results, shorter pain duration. | High | **Public Acceptance** | Low | Slower process, longer waiting time, and more painful than fingerstick testing; may conflict with cultural or traditional beliefs. |
| Needle depth depends on individual skin sensitivity. | Simple | **Ease of testing** | Difficult | Overweight individuals or those with thick subcutaneous fat may present difficulties in locating veins. |
| Requires tool familiarity and practice. | Difficult | **Operational familiarity** | Simple | Easy for medical staff with experience in blood collection to get started. |
